# Gender norms and responsibility for preconception health improvement: challenges and recommendations for action from the 2024 UK preconception EMCR network conference

**DOI:** 10.1186/s12978-026-02402-0

**Published:** 2026-06-30

**Authors:** Merissa Elizabeth Hickman, Manjiri Khare, Danielle Schoenaker, Madeleine Benton, Emma H Cassinelli, Kriss Fearon, Stephanie J. Hanley, Shivali Lakhani, Catherine Stewart, Michael Daly

**Affiliations:** 1 Population Health Sciences, University of Leicester, Leicester, UK; 2 University Hospitals of Leicester, Leicester, UK; 3 School of Human Development and Health, Faculty of Medicine, University of Southampton, Southampton, UK; 4 MRC Lifecourse Epidemiology Centre, University of Southampton, Southampton, UK; 5 National Institute for Health and Care Research (NIHR) Southampton Biomedical Research Centre, University of Southampton and University Hospital Southampton NHS Foundation Trust, Southampton, UK; 6 Department of Psychological Medicine, King’s College London, London, UK; 7 School of Medicine, Dentistry and Biomedical Sciences, Queen’s University Belfast, Belfast, Northern Ireland, UK; 8 Centre for Reproduction Research, De Montfort University, 0.23 Edith Murphy House, The Gateway, Leicester, UK; 9 Institute of Applied Health Research, University of Birmingham, Birmingham, UK; 10 Robert Gordon University, Garthdee Road, Aberdeen, UK; 11 Reproductive Health, Institute for Women’s Health, University College London, London, UK; 12 Population Health Sciences, University of Bristol, Bristol, UK

## Abstract

Preconception health improvement is vital for maternal and child health, yet societal perceptions about who holds responsibility for this remain underexplored. We conducted a hybrid workshop at the 2024 UK Preconception Early-Mid Career Researcher (EMCR) Network conference, attended by academics, health professionals and members of the public (*N* = 60). Small-group discussions on responsibility for preconception health improvement were audio-recorded and transcribed alongside notes from online whiteboards and paper-based flipcharts. Using framework analysis, we applied a deductive coding framework derived from the workshop questions. Attendees felt a disproportionate burden of responsibility for preconception health improvement was placed on women, reflecting deeply entrenched gendered norms within research, healthcare and broader societal structures. Attendees’ recommendations centred around (i) society, community and culture, (ii) education and early awareness, (iii) considerations for future research, and (iv) systems-level and policy solutions. These findings provide valuable insights for developing equitable public health strategies and research agendas relating to preconception health.

## Abstract (plain language summary)

Being healthy before pregnancy and parenthood is important. It affects pregnancy outcomes and lifelong wellbeing. But there are mixed views on who should be responsible for improving health before pregnancy. We held a workshop with 60 people, including health workers, researchers, and members of the public. We asked who is responsible for health before pregnancy and how this shows up in different contexts. We also asked for suggestions for how to make sure this responsibility is shared evenly.

Attendees felt that women carry most of the burden. This happens in health care, research, and everyday life. Men are often left out of conversations about health around pregnancy. Current approaches to improving health before pregnancy focus on women’s choices and actions such as diet and smoking rather than wider issues like poverty, education, and health care access.

Attendees felt that health before pregnancy should be a shared responsibility between partners, health workers, government, and society. They suggested including men equally in health care, research, and campaigns around health before pregnancy. They also said that education about this should start early, in schools. They called for changes in communities to support health before pregnancy, so that people don’t need to do this alone.

These suggestions can help create fairer approaches to improving health before pregnancy and parenthood that support everyone. They reflect that this requires support from the whole of society.

## Introduction

Preconception health has become an increasingly important focus of public health research and policy, reflecting growing recognition that health before pregnancy can influence pregnancy outcomes as well as the lifelong well-being of future generations [11, 24]. The preconception period includes all reproductive years when a person’s overall health, lifestyle and environmental exposures can influence future perinatal outcomes [24]. This perspective has supported the alignment of preconception health improvement with broader agendas in preventative medicine, reproductive justice and health inequalities. Whilst there is no universally accepted definition for preconception health, some adopt a narrow biomedical focus, prioritising women’s health immediately prior to conception [25]. Conversely, others take a more comprehensive approach, accounting for a wider range of health behaviours, environmental exposures, and social determinants relevant to both women and men [21, 28]. These divergent definitions are not neutral; they reflect and reinforce wider assumptions about gender and responsibility for reproductive health improvement.

Academic sociologists have criticised preconception health initiatives for centring on women’s bodies and behaviours as the primary site of intervention [2, 4, 27]. This criticism is supported by the focus of the current epidemiological literature; a recent umbrella review found that under 7% of reported associations between preconception exposures and perinatal outcomes related to paternal exposures [11], suggesting these are understudied and reported. However, the limited literature on paternal preconception risk factors suggests that factors including paternal age, obesity and health behaviours are associated with adverse maternal and child health outcomes [5, 9, 15]. This highlights that an exclusive focus on women in the preconception period risks overlooking important opportunities to reduce the risk of adverse perinatal outcomes. Other studies have identified gaps in services and interventions addressing men’s reproductive health, highlighting a need for more inclusive strategies that move beyond maternal-centric models [1, 22]. Similarly, a recent scoping review of grey literature found that preconception advice continues to be directed primarily towards women, particularly those with pre-existing health conditions, while guidance for men and same-sex partners remains limited [6]. Authors have suggested that this overfocus on maternal contributions can manifest as maternal self-blame for adverse outcomes [14, 27].

In prior qualitative studies, women have reported diverse perspectives on responsibility for preconception health improvement. Some have expressed a desire for an increased focus on paternal health behaviours and education, noting the disproportionate responsibility placed on mothers-to-be for ensuring a healthy pregnancy [18] and that both birth parents are ‘equally responsible’ for their child’s health [10]. These women have also called for more interventions addressing the wider, socioeconomic determinants of preconception health [10]. Conversely, women in other studies have expressed that it is women’s responsibility to prepare for pregnancy and childbirth [19] and that encouraging individual responsibility for this is more impactful than ‘government interference’ through interventions [10]. While recent work has explored healthcare professionals’ perspectives on preconception care (e.g. Caut et al., [8], the views of other key stakeholders and academic disciplines beyond sociology remain underexplored. This is a key evidence gap to address, as understanding how key stakeholder groups view and conceptualise this responsibility may help to identify, and develop strategies to address, missed opportunities for preconception health improvement arising from misaligned stakeholder expectations. To address this gap, we held an interactive workshop at the 2024 UK Preconception Early-and Mid-Career Researchers (EMCR) Network conference involving academic researchers, healthcare professionals, policymakers and members of the public. There is also a lack of clear guidance on how responsibility for preconception health improvement should be shared. To address this gap, we held an interactive workshop at the 2024 UK Preconception Early-and Mid-Career Researchers (EMCR) Network conference involving academic researchers, healthcare professionals, policymakers and members of the public. This paper reports their views on societal norms around responsibility for improving preconception health, whether these norms are gendered and how they manifest in different contexts, and their recommendations for ensuring responsibility for preconception health is more evenly shared.

## Methods

### Workshop

The UK Preconception EMCR Network conference 2024 was a one-day hybrid event in November 2024 in Leicester, England. We held a 75-minute, interactive workshop titled ‘Gender norms and responsibility for preconception health improvement’. Sixty conference attendees participated in the workshop (*N* = 60), including academic researchers (*n* = 40), health professionals (*n* = 10), members of the public (*n* = 6), and attendees from other sectors (*n* = 4). These classifications are based on primary affiliations; several attendees held dual roles across academia, clinical practice, and third-sector or private organisations. Attendees’ expertise spanned preconception health and care, maternal and child health, sexual and reproductive health, obstetrics and gynaecology, primary care, public health, psychology, nutrition, and epidemiology. MEH, MPD and MK introduced the workshop with a brief presentation covering conflicting definitions of preconception health and care, contrasting views on responsibility for preconception health improvement, and a relevant clinical case scenario. Attendees were then asked to consider and discuss three sets of questions, which were developed through co-author group discussion to address the evidence gaps outlined in the introduction:

1. Who does society consider to be responsible for preconception health improvement? In what ways?
2. Is there a gendered element to who society holds responsible for preconception health improvement? How does this manifest in healthcare, academic research, and wider society?
3. What are your recommendations to ensure responsibility for preconception health improvement is shared evenly? Who are these recommendations for?

These questions were designed to explore how responsibility for preconception health improvement is currently understood and distributed, while allowing space for participants to challenge or reject their underlying assumptions.

Attendees were pre-assigned to small groups, to ensure a range of backgrounds and perspectives within each group (*n* = 4 groups online, *n* = 4 in person), and recorded their discussion points on flipchart paper or Zoom Whiteboards. Designated spokespeople from each group subsequently relayed these points to the wider group whilst being audio-recorded. As such, the collected data represent group-level accounts rather than individual perspectives.

### Analysis

Data were analysed using the framework method [13], an approach well-suited to meeting pre-established objectives in applied qualitative research whilst also allowing novel concepts to be inductively derived from the data [3]. The workshop recordings were transcribed verbatim and uploaded to the NVivo 14 software package, along with the notes attendees added to their flipchart paper or Zoom Whiteboard. MEH and MD familiarised themselves with the material and added deductive code labels, corresponding to the three workshop questions, to all relevant excerpts. Inductive codes were developed from the transcripts, which MEH and MD coded in duplicate. They charted the data into matrices using Microsoft Excel, paraphrasing coded excerpts and including illustrative quotations. Candidate themes were developed by identifying data patterns, selected based on the study’s aims, deductive codes and inductively derived concepts, and elaborated through analytical memos [13]. We re-read the transcripts to ensure these formed a coherent narrative of the data and answered the research questions. All authors met as a group to discuss, refine and agree on final themes and subthemes. The finalised themes and subthemes are reported as an analytic narrative. To enhance analytical depth and support interpretation, the analysis was informed by the socio-ecological model of health, which was used as a sensitising framework to organise and interpret the recommendations across the model’s individual, interpersonal, organisational, and policy/system levels [12]. The use of the terms ‘men’ and ‘women’ throughout the analytic narrative primarily relates to cisgender men and women in heterosexual partnerships, reflecting how attendees framed their discussions.

## Results

### Who society considers responsible for preconception health improvement

Attendees highlighted how societal norms, cultural expectations, and institutional practices reinforce gender norms and overwhelmingly frame reproduction as a “woman’s issue”, with women being primarily responsible for reproductive health improvement and planning. This framing was perceived as deeply embedded across multiple sectors — including healthcare, education, and media — as well as public discourse:

*“Societal responsibilities*,* through the preconception*,* pregnancy and parenthood journey*,* tend to focus on women…"*.

*“Everybody sees it [preconception health improvement] as a woman’s responsibility… if she doesn’t do everything… she feels bad about everything.”*

This was felt to include the burden for securing contraception, navigating fertility, and improving preconception health:

*“Preconception is perceived as a gender-based concept… women are expected to be responsible.”*

Conversely, attendees reported that men are frequently seen as secondary or excluded from reproductive decisions and conversations, with their reproductive roles overlooked or undervalued by both society and healthcare systems.

*“Men often feel sort of secondary in the decision to have children.”*

Attendees also noted that current approaches overwhelmingly focus on individual — usually maternal — behaviours, such as diet and physical activity, rather than recognising the broader socio-ecological factors influencing reproductive health. They felt this status quo is problematic as individual behaviour change alone is not sufficient to fully optimise preconception health, and must be supported by health-promoting environments, community initiatives, and government policy:

*“Responsibility tends to fall on the individual and females*,* currently*,* but the social ecological model shows behaviour can’t change without support from infrastructure*,* policy*,* and healthcare.”*

*“Everyone plays a role across the layers of society — behaviour can’t happen or be sustained in isolation.”*

Healthcare services, including primary care, and early, ongoing health education were identified as critical in enabling informed decisions and sustaining preconception behaviour change:

*“Health education from a young age is critical to set people up for understanding preconception health.”*

### Manifestations of gendered societal norms around preconception health improvement

#### Academic research

Attendees felt that academic research on preconception health is shaped and limited by gendered assumptions that prioritise women’s roles and perspectives while marginalising men. This was seen to influence both the scope and framing of research, often reducing complex social phenomena to individual — typically maternal — factors. Cultural and social dimensions, as well as paternal contributions to perinatal and developmental outcomes, were felt to be underexplored:

*“Most of the literature*,* especially in epidemiology*,* is really focusing on the role of the woman*,* but it would be interesting to focus back more of this research on the role of the men*,* or phrasing it in such a way that it’s not just the woman being exposed to something*,* but it’s the family unit [that’s important]”.*

*“Look at academic research… we know very little about male [preconception health] indicators and what impact that has on outcomes… very few interventions [are] being targeted [at men].”*

Attendees noted that the “difficulty” of recruiting men into preconception health research reflects deeper systemic and cultural exclusion and necessitates new recruitment methods and inclusive approaches.

#### Healthcare systems

Attendees described a consistent tendency within healthcare systems, clinical interactions, and health communications to prioritise women in discussions about reproductive and preconception health. This focus was felt to shape who receives information, who is held accountable for reproductive outcomes, and how care pathways are structured:

*“Health messaging around preconception health and pregnancy is usually [focused on] women… [it’s] deeply ingrained.”*

*“Often women are the ones referred to check for health and fertility issues… rather than checking with men.”*

Men’s reproductive health was felt to be overlooked in consultations, particularly those relating to contraception and fertility. Healthcare was also seen as biased in how questions are directed and to whom services are tailored, reinforcing the perception that reproductive health is a woman’s domain:

*“Women take on [the] stigma of male infertility.”*

### Recommendations

Attendees advocated for extending responsibility for preconception health improvement beyond individuals - particularly women - to a wider group including healthcare professionals, government, educational institutions, and society at large. They called for a shift from the current woman-centred model to a more equitable approach that involves the equal inclusion of men, challenges gendered assumptions and the current hyper-focus on “*the role of the woman*” and considers system-wide strategies. This reflected their view that preconception health improvement requires a shared, systemic approach involving coordinated efforts across multiple sectors and layers of society and should therefore be seen as a collective responsibility involving individuals, healthcare providers, public health bodies, educators, and policymakers.

*“Everyone should be responsible for improving preconception health.”*

*“Who’s responsible? Essentially*,* everybody has a role to play.”*

Table 1 shows that attendees’ recommendations related to different aspects of the change needed to achieve more equitable preconception health approaches. Importantly, the recommendations focused on community-, organisational-, and policy-level solutions, suggesting that attendees place greater value on structural approaches rather than individual behaviour change alone. These recommendations ranged from immediate practical changes within healthcare settings to broader cultural and policy transformations that challenge deeply embedded gender norms around reproductive responsibility. A central theme across all recommendations was the need to fundamentally reframe preconception health as a collaborative endeavour rather than an individual responsibility. Attendees consistently emphasised moving toward what they described as a “team effort” or “[family] unit approach” that recognises the shared nature of reproductive decisions and outcomes. This shift requires coordinated action across multiple levels, from individual healthcare consultations to national policy frameworks.

**Table 1:** Participant recommendations for encouraging societal responsibility for preconception health improvement

Socio-ecological domain (context)	Recommendations	Key participant quotes
1. Community (culture)	i. Move toward a collaborative, collective approach ii. Use gender-neutral language iii. Normalise male involvement in reproductive healthcare and societal discourse iv. Frame preconception health improvement as a shared concern v. Promote open, honest conversations between healthcare professionals and reproductive-aged patients vi. Challenge cultural norms around gendered reproductive responsibility	*“Make the pregnancy a team effort*,* and take away the focus from the role of the woman… focus it towards the role of the [family] unit.”* *“Increase knowledge about the role men have and the impact their health can have.”* *“Pregnancy should be seen as a collaboration.”* *“Cultural norms… promote open*,* honest conversations with healthcare professionals and peers.”*
2. Organisational level (education)	i. Embed preconception health in school curricula, and medical and midwifery training ii. Update professional knowledge post-qualification iii. Include preconception education in school sex education iv. Target people of all genders v. Utilise social media influencers	*“Embed preconception in education - school age*,* medical training*,* midwifery.”* *“Public health messaging*,* TikTok creators… not only targeted to women.”* *“Use gender-neutral information so both partners are aware.”*
3. Organisational level (research)	i. Include more men in preconception health research and as patient & public involvement representatives ii. Develop couple-based research approaches iii. Focus on the influence of paternal health iv. Challenge woman-centric research	*“More focus on men in research… include a broader range of people in decision making.”* *“More research on the father’s health"* *“Making research on everyone as default unless there is specific justifications otherwise”*
4. System / Policy	i. Create specific reproductive health “touch points” for men under 40 years ii. Train healthcare professionals in male preconception health iii. Initiate conversations with both birth parents equally iv. Ask men about their family planning intentions* & include more men in preconception health research v. Develop couple-based research approaches vi. Challenge woman-centric research defaults vii. Include a broader range of attendees in decision-making	*“GPs proactively calling men in [for preconception counseling]… normalising that process.”* *“Men [should be] asked whether they would like to have a child…potentially by a GP.”* *“Healthcare professionals initiating conversations with both men and women.”* *“More research on the father’s health"*

*Patients’ pregnancy intentions are not routinely queried in UK healthcare appointments

These recommendations suggest transforming preconception health from its current woman-centric focus toward a more equitable approach. Attendees recognised that achieving this transformation requires sustained effort across multiple domains, with particular emphasis on addressing the structural and cultural barriers that currently exclude men from reproductive health conversations and decision-making processes.

The urgency of these recommendations is underscored by attendees’ recognition that current approaches not only fail to optimise preconception health outcomes but also perpetuate harmful gender inequalities that place disproportionate burden and responsibility on women. Their vision for change encompassed both immediate practical steps that could be implemented within existing systems, and longer-term cultural shifts that challenge fundamental assumptions about reproductive responsibility and gender roles.

## Discussion

Preconception health improvement is vital for maternal and child health, yet societal perceptions about who holds responsibility for this remain underexplored. To our knowledge, this is the first qualitative study to explore views on responsibility for preconception health improvement, how this manifests in society and how to ensure it is evenly shared, with a mixed group of healthcare professionals, academic researchers and members of the public. It is important to acknowledge that it was not possible to attribute individual quotes to specific stakeholder groups as feedback was provided collectively via designated spokespersons representing each discussion group. As such, the data reflect group-level positions rather than discrete individual perspectives. While this approach facilitated the capture of shared viewpoints and areas of consensus, it limited our ability to explore potential differences in perspectives across stakeholder categories (e.g., healthcare professionals, researchers, policymakers). This should be considered when interpreting the findings, as important nuances between different stakeholders may not be fully captured.

Attendees highlighted a need for a more collective, society-wide approach to preconception health improvement, acknowledging that individual behaviour change must be supported by health-promoting environments and policies. They also highlighted a need for further research on the wider determinants of preconception health and paternal preconception risk factors, and a need to include more men in preconception health research to reflect this.

### Integration with prior research

Attendees’ views echoed longstanding critiques from sociology and public health, which argue that preconception health initiatives too often centre on heterosexual, cisgendered women’s bodies and behaviours while marginalising paternal contributions and structural determinants [2, 4, 27]. Epidemiological evidence also reflects this imbalance; fewer than 7% of reported associations between preconception exposures and perinatal outcomes concern paternal factors, and there is a gap in the examination of the wider determinants of preconception health, such as pollution, poverty and abusive relationships [11]. Attendees’ call for more inclusive approaches, greater recognition of wider determinants, and broader system-level support aligns with emerging international perspectives that advocate holistic, equity-focused strategies engaging both men and women, families, and diverse communities [28, 29]

Importantly, some scholars have argued that the term ‘preconception health’ itself may reinforce maternal responsibility and individualised accountability [26]. Attendees’ reflections resonate with this critique: current discourse and messaging in preconception health frequently position women as the locus of responsibility, while minimising men’s roles and structural influences. Reframing language and interventions to reflect shared responsibility and reproductive justice may therefore be essential to achieving equitable progress. Moreover, attendees’ call for more focus on the wider determinants of health is supported by epidemiological analyses and recent evidence syntheses, which suggest that socio-economic and environmental factors are significant predictors of preconception health and reproductive outcomes, with disparities magnified by gender and other axes of inequality [16, 28, 29]. These findings align with recommendations developed at the 2023 UK Preconception EMCR Network workshop, which similarly emphasized the need for multi-level interventions addressing structural determinants alongside individual behaviours [23].

### Implications

Positioning women as primarily accountable for preconception health risks overlooking opportunities to optimise paternal health, reinforces gendered inequities, and places undue pressure on women to manage risks largely shaped by structural conditions such as poverty, housing, employment and healthcare access [15]. Our findings suggest the need for intersectional and equity-focused approaches that take account of macro-level determinants and community contexts which shape preconception health. There is also a need for early and ongoing health education, delivered to all genders, to help normalise the idea of shared responsibility for reproductive health. Healthcare systems should adopt more inclusive practices that acknowledge and support paternal as well as maternal health. Finally, further research is required to interrogate entrenched gender norms and to prioritise structural as well as behavioural determinants of preconception health.

Mirroring findings that there is a lack of consideration for sexual and gender minorities in preconception health guidance, policies, strategies and clinical practice [7, 17, 20], there was an absence of discussion regarding diverse gender and sexual identities at the workshop. Throughout the discussions, attendees’ reflections centred almost exclusively on cisgender men and women in heterosexual relationships planning conception. LGBTQ+ families, non-binary people, and alternative pathways to parenthood (such as surrogacy, adoption, or assisted reproductive technologies involving donors) were not raised. This indicates that heteronormative assumptions about reproduction are deeply ingrained even among healthcare professionals and researchers working in reproductive health. It suggests that challenging gendered norms around preconception health requires more than examining the unequal distribution of responsibility between cisgender men and women. It also requires broadening preconception health frameworks to include gender-diverse, LGBTQ+, and non-traditional reproductive experiences. Future research should address this gap to ensure more inclusive preconception health improvement strategies. A limitation of the workshop is that we did not prompt attendees to consider minority sexual and gender groups in their discussions.

## Conclusion

Preconception health improvement is widely recognised as critical for maternal, child, and long-term population health, but current approaches remain overly individualised and gendered. Our findings demonstrate how women continue to be framed as primarily responsible for reproductive health improvement and outcomes, while men’s roles and contributions to perinatal outcomes are overlooked. Attendees called for a shift toward collective, system-level approaches that distribute responsibility more fairly and recognise the influence of broader social, economic, and environmental contexts. Only through such comprehensive reframing can preconception health policy and practice achieve improved equity in optimising health outcomes for current and future generations.

## Data Availability

All data supporting the findings of this study are available within the paper and its Supplementary Information.

## References

[1] Agustina SA, Prabandari YS, Hakimi M, Hayati EN. How to Engage Men in Preconception Health? A Scoping Review. Iran J Nurs Midwifery Res. 2024;29(6):660–8. 10.4103/ijnmr.ijnmr_27_23. 39759910 PMC11694592

[2] Almeling R, Waggoner MR. More and Less than Equal: How Men Factor in the Reproductive Equation. Gend Soc. 2013;27(6). 10.1177/0891243213484510. PMC3856903 24347818

[3] Braun V, Clarke V. Conceptual and Design Thinking for Thematic Analysis. Qualitative Psychol. 2021;9(1). 10.1037/qup0000196.

[4] Budds K. Fit to conceive? Representations of preconception health in the UK press. Feminism Psychol. 2021;31(4). 10.1177/0959353520972253.

[5] Carter T, Schoenaker D, Adams J, Steel. Paternal preconception modifiable risk factors for adverse pregnancy and offspring outcomes: a review of contemporary evidence from observational studies. BMC Public Health. 2023;23(1). 10.1186/s12889-023-15335-1. PMC10022288 36927694

[6] Cassinelli EH, McKinley MC, Kent L, Eastwood KA, Schoenaker D, Trew D, Stoikidou T, McGowan L. Preconception health and care policies, strategies and guidelines in the UK and Ireland: a scoping review. BMC Public Health. 2024;24(1):1662. 10.1186/s12889-024-19188-0 . PMID: 38909211; PMCID: PMC11193169. 38909211 PMC11193169

[7] Cassinelli EH, Kent L, Eastwood K, Schoenaker D, McKinley MC, McGowan L. Preconception Health Indicators and deprivation: A cross-sectional study using National Maternity Healthcare Data. BJOG: Int J Obstet Gynecol. 2025;132(13):2138–48. 10.1111/1471-0528.18256. PMC12592756 40536104

[8] Caut C, Schoenaker D, McIntyre E, et al. Health professionals’ beliefs and attitudes towards preconception care: a systematic review. BMC Health Serv Res. 2025;25:1023. 10.1186/s12913-025-13246-y. 40760480 PMC12323077

[9] Daly MP, White J, Sanders J, Kipping RR. Women’s knowledge, attitudes and views of preconception health and intervention delivery methods: a cross-sectional survey. BMC Pregnancy Childbirth. 2022;22(1):729. 10.1186/s12884-022-05058-3. PMID: 36151510; PMCID: PMC9508727. PMC9508727 36151510

[10] Daly MP, Kipping RR, White J, Sanders J. What support is needed for preconception health improvement, and by whom? A qualitative study of women’s views. 2025. 10.1101/2024.12.04.24318497.

[11] Daly M, Kipping RR, Tinner LE, Sanders J, White JW. Preconception exposures and adverse pregnancy, birth and postpartum outcomes: Umbrella review of systematic reviews. Paediatr Perinat Epidemiol. 2022;36:288–99. 10.1111/ppe.12855. 34970757

[12] Ewald B, McKenzie S, Harris M. The path to good health: Shifting the dialogue and promoting social ecological thinking. Aust J Prim Health. 2023;29(3):191–4. 10.1071/PY22220. PMC10041553 36992717

[13] Gale NK, Heath G, Cameron E, Rashid S, Redwood S. Using the framework method for the analysis of qualitative data in multi-disciplinary health research. BMC Med Res Methodol. 2013;13(1). 10.1186/1471-2288-13-117. PMC3848812 24047204

[14] Gold KJ, Sen A, Leon I. Whose Fault Is It Anyway? Guilt, Blame, and Death Attribution by Mothers After Stillbirth or Infant Death. Illn Crisis Loss. 2018;26(1):40–57. 10.1177/1054137317740800.

[15] Huang JY, Low FM, Kee MZL, Hopper LN, Sum KK, Chung GSK, Kaholokula JK, Stephenson J, Schoenaker D, Godfrey KM. More equitable preconception health: paternal lifecourse opportunities for better pregnancy, child, and family outcomes. Lancet. 2026. 10.1016/S0140-6736(26)00148-0. 41856159

[16] Khekade H, Potdukhe A, Taksande AB, Wanjari MB, Yelne S. Preconception Care: A Strategic Intervention for the Prevention of Neonatal and Birth Disorders. Cureus. 2023. 10.7759/cureus.41141. 37519532 PMC10386873

[17] Limburg A, Everett BG, Mollborn S, Kominiarek MA. Sexual Orientation Disparities in Preconception Health. J Women’s Health. 2020;29(6). 10.1089/jwh.2019.8054. PMC7307698 32105564

[18] McGowan L, Lennon-Caughey E, Chun C, McKinley M, Woodside J. Exploring preconception health beliefs amongst adults of childbearing age in the UK: A qualitative analysis. BMC Pregnancy Childbirth. 2020;20(1). 10.1186/s12884-020-2733-5. PMC6966858 31948431

[19] M’hamdi HA, Sjpkens MK, Beaufort I, Rosman AN, Steegers EAP. Perceptions of pregnancy preparation in women with a low to intermediate educational attainment: a qualitative study. Midwifery. 2018;59(1):62–7. 10.1016/j.midw.2018.01.004. 29396381

[20] Permezel J, Arnold ASC, Thomas J, Maepioh AL, Brown R, Hafford-Letchfield T, Skouteris H, Hatzikiriakidis K, McNair RP. Experiences in the delivery of preconception and pregnancy care for LGBTIQA+ people: A systematic review and thematic synthesis of patient and healthcare provider perspectives. Midwifery. 2023;123. 10.1016/j.midw.2023.103712. 37178659

[21] Public Health England. Preconception care: Making the case. 2018. Available at: https://www.gov.uk/government/publications/preconception-care-making-the-case. Accessed: 30 Dec 2025.

[22] Rabiei Z, Shariati M, Mogharabian N, Tahmasebi R, Ghiasi A, Motaghi Z. Men’s Knowledge of Preconception health: A systematic review. J Family Med Prim Care. 2023;12(2):201–7. 10.4103/jfmpc.jfmpc_1090_22. 37091006 PMC10114563

[23] Schoenaker D, Hall J, Stewart C, Hanley SJ, Cassinelli EH, Benton M, Wynn-Jones AA, Chawla M, Currie S. 2023 UK Preconception EMCR Network conference workshop attendees. Tackling inequalities in preconception health and care: barriers, facilitators and recommendations for action from the 2023 UK preconception EMCR network conference. J Dev Orig Health Dis. 2024;15:e24. 10.1017/S204017442400031X. PMID: 39444318. 39444318

[24] Stephenson J, Heslehurst N, Hall J, et al. Before the beginning: nutrition and lifestyle in the preconception period and its importance for future health. Lancet. 2018;391(10132):1830–41. 29673873 10.1016/S0140-6736(18)30311-8 PMC6075697

[25] US Office on Women’s Health. Preconception Health | Office on women’s health. 2021. Available at: https://womenshealth.gov/pregnancy/you-get-pregnant/preconception-health. Accessed: 30 Dec 2025.

[26] Waggoner M, Pentecost M. The limits of preconception care for global health. Sexual and Reproductive Health Matters; 2025. 10.1080/26410397.2025.2499329 PMC12107648 40296845

[27] Waggoner MR. (2017). The zero trimester: Pre-pregnancy care and the politics of reproductive risk. In *The Zero Trimester: Pre-Pregnancy Care and the Politics of Reproductive Risk*. 10.3138/ijfab.12.2.br01

[28] Welshman H, Dombrowski S, Grant A, Swanson V, Goudreau A, Currie S. Preconception knowledge, beliefs and behaviours among people of reproductive age: A systematic review of qualitative studies. Prev Med. 2023. 10.1016/j.ypmed.2023.107707. 37730135

[29] World Health Organisation [WHO]. (2024). Reframing care and services to improve preconception health: meeting report, Geneva, Switzerland, 8–9 May 2024. Geneva.

